# Potential role of stress granules and myogranules in amyotrophic lateral sclerosis

**DOI:** 10.3389/fnmol.2025.1686230

**Published:** 2026-01-05

**Authors:** Saddam Muhammad Ishaq, Aaron P. Russell

**Affiliations:** Institute for Physical Activity and Nutrition, School of Exercise and Nutrition Sciences, Deakin University, Geelong, VIC, Australia

**Keywords:** amyotrophic lateral sclerosis, brain, myogranule, skeletal muscle, spinal cord, stress granules, TDP-43

## Abstract

Amyotrophic lateral sclerosis (ALS) is characterized by the progressive loss of upper and lower motor neurones, leading to muscle wasting, paralysis and respiratory failure. Pathological cytoplasmic aggregation of the RNA-binding protein transactive response DNA-binding protein 43 (TDP-43) protein occurs in neural tissues in ~97% of all ALS cases, and is also observed in skeletal muscle. Cytoplasmic aggregation of TDP-43 is believed to contribute to ALS pathogenesis; however, its precise mechanistic role/s continues to elude the field. This mini review explores the potential role and regulation of two TDP-43-associated RNA-protein assemblies, stress granules (SGs) and myogranules (MGs). We review the current understanding of SG and MG formation and their potential role in ALS-related neurodegeneration and muscle pathology. We also highlight limitations and strengths and suggest future directions for research.

## Introduction

Amyotrophic lateral sclerosis (ALS) is a neurodegenerative disease characterized by motor neurone (MN) degeneration, muscle atrophy, paralysis, and eventual respiratory failure (Brown and Al-Chalabi, 2017). The minority of ALS cases are familial (FALS; 5–10%). The predominant known gene mutations are found in the chromosome 9 open reading frame 72 (C9orf72), superoxide dismutase type 1 (SOD1), fused in sarcoma (FUS), ataxin-2 (ATXN2) and TAR DNA binding protein (TARDBP) genes (Andersen and Al-Chalabi, 2011). The majority of cases are sporadic (SALS; 90–95%) with no known cause (Andersen and Al-Chalabi, 2011). Independent of disease etiology, common disease-modifying pathways include, but are not limited to, alternative splicing, nucleo-cytoplasmic trafficking (Simona et al., 2020), axonal transport, and RNA metabolism (Fan and Leung, 2016). Abnormalities in RNA metabolism appear to be a major mechanistic contributor to ALS and are linked to the most common pathological characteristic, which is the cytoplasmic mislocalization and aggregation of TAR DNA binding protein (TDP-43) that occurs in about 97% of all cases (Ling et al., 2013; Neumann et al., 2006). TDP-43 protein aggregation is observed in multiple tissues, including the brain, spinal cord and skeletal muscle (Cykowski et al., 2018; Ling et al., 2013; Mori et al., 2019; Neumann et al., 2006; Tsitkanou et al., 2022), indicating a pathological effect that extends beyond MNs. This is consistent with the more general histopathological features of ALS, which, independent of disease etiology, are pathologically characterized by both upper and lower MN degeneration in the brain and spinal cord with skeletal muscle wasting.

## TAR DNA-binding protein 43 (TDP-43)

In a healthy cell, the prominent physiological roles of TDP-43 in the nucleus involve RNA regulation, including stability of mRNA, transcriptional regulation, transport, and alternative splicing (Geuens et al., 2016; Nakielny and Dreyfuss, 1997; Scotter et al., 2015). In the cytoplasm of cells within neural and skeletal tissue, TDP-43 participates in protein translation, transport granule formation, and RNA granule formation, including stress granules (SGs) and myogranules (MGs) (Prasad et al., 2019; Vogler et al., 2018). During cellular stress in neural tissue, TDP-43 helps control protein translation and promotes cellular recovery through the formation of SGs (Lee et al., 2022). In muscle tissue, TDP-43 plays a regulatory role in mRNA localisation and translation by forming myogranules. MGs are RNA-protein assemblies enriched with mRNAs associated with sarcomere formation that support muscle regeneration and development (Vogler et al., 2018). However, under pathological conditions, such as ALS, TDP-43 accumulates and aggregates in the cytoplasm of the brain and spinal cord (Arai et al., 2006; Mitchell et al., 2015; Neumann et al., 2006) and has been observed to aggregate in skeletal muscle (Tsitkanou et al., 2022). How cytoplasmic TDP-43 accumulation occurs and contributes to ALS pathogenesis continues to elude the field. The involvement of TDP-43 in granule formation, specifically SGs and MGs, and their dynamic change from physiological to pathological entities, may be a factor contributing to MN degeneration. It is important to note that while SGs and MGs are frequently observed in cells undergoing pathological changes, their presence does not necessarily imply causation. The difference between correlation and causality remains a central challenge in neurodegeneration research. SG formation may reflect a cellular response to stress rather than a direct driver of pathology. However, persistent or aberrant SG dynamics, particularly in the context of ALS-linked mutations, may disrupt RNA metabolism and protein homeostasis, thereby contributing to disease progression.

## Stress granules and their composition

Stress granules (SGs) are dynamic and transient structures made up of RNA and protein. They are membrane-free organelles that form in the cytoplasm of cells in response to stress (Kedersha and Anderson, 2007; Kedersha et al., 2005; Ohn et al., 2008). SG formation begins when translation stops, which is usually caused by the phosphorylation of eIF2α. This causes polysomes to break down and mRNAs and RNA-binding proteins (RBPs) to build up around stalled ribosomes and endoplasmic reticulum (ER) membranes. These areas function as nucleation sites where spatially limited biochemical signaling and molecular crowding result in high local concentrations of vital proteins and RNAs (Cui et al., 2024; Wolozin and Ivanov, 2019).

SG formation may occur through liquid–liquid phase separation (LLPS), a biophysically defined process driven by multivalent interactions among RBPs that contain intrinsically disordered regions (IDRs) and prion-like domains. These domains promote condensation into liquid-like assemblies at specific cytoplasmic locales (Cui et al., 2024; Lin et al., 2015). The role of LLPS in SG formation and disease pathology is of considerable interest. However, LLPS has methodological limitations and interpretive challenges. In vitro studies offer controlled environments for manipulating variables such as protein concentration and pH and have successfully reconstituted LLPS using purified proteins like FUS, TDP-43, and G3BP1 (Fernandes et al., 2018). However, these systems may oversimplify cellular complexity and produce false positives under artificial conditions (Hedtfeld and Musacchio, 2024). Live cell studies provide physiological relevance of SGs, revealing functional roles through genetic manipulation (Yuan et al., 2025). However, these also lack the complex interplay of multiple cells and processes seen *in vivo*.

SG formation may also involve mechanisms beyond LLPS, such as percolation, cytoskeletal transport, RNA scaffolding, and post-translational modifications (PTMs), all of which are implicated in neurodegenerative disease pathology (Buchan, 2024; Campos-Melo et al., 2021; Wolozin and Ivanov, 2019). For instance, cytoskeletal elements like microtubules and actin filaments regulate SG positioning and clearance (Böddeker et al., 2023), while RNA molecules act as scaffolds that recruit RNA-binding proteins (RBPs) to nucleate SGs (Campos-Melo et al., 2021). PTMs such as phosphorylation and glutathionylation can modulate the phase behavior of SG proteins and are disrupted in ALS and FTD (Choi et al., 2023). Computational models like PSPredictor and Droppler enable high-throughput screening and identification of LLPS-prone sequences (Wang et al., 2021), though they lack environmental biological context. Therefore, claims, such as LLPS being a direct cause of disease or that all SGs form via LLPS, need to be taken with caution (Li et al., 2024; Zhang et al., 2024). Standardized assays, multi-modal validation, and integrative models that incorporate material state transitions and cellular signaling are needed to advance our understanding of the mechanisms causing SG formation, SG composition and their role in disease (Spruijt, 2023; Yuan et al., 2025).

The proteins and RNA cargo within SGs remain dynamic, moving between the granules and the cytoplasm (Feric et al., 2016; Shin and Brangwynne, 2017). mRNAs are present in SGs, highlighting SG formation as a regulator of RNA metabolism. SGs are also closely associated with translation and the sorting of specific mRNAs for different fates (Kedersha and Anderson, 2009). Their physiological formation is a protective mechanism, temporarily conserving energy and maintaining cellular homeostasis during stress, with their dissipation occurring post the stress event (Collier and Schlesinger, 1986; Glineburg et al., 2024). However, chronic stress or pathological conditions may lead to dysregulated SG dynamics (Wolozin and Ivanov, 2019). This may result in persistent protein accumulation or aggregation within SGs, impairing normal protein function and potentially causing cellular dysfunction (Anderson and Kedersha, 2009; Jeon and Lee, 2021; Mahboubi and Stochaj, 2017; Riggs et al., 2020). Sustained formation or impaired dissipation of SGs is implicated in diseases including cancer, viral infection and neurodegenerative disorders (Ash et al., 2014; Choi et al., 2019; Panas et al., 2012; Taylor et al., 2016).

The composition of SGs is influenced by different stressors and cellular environments (Anderson and Kedersha, 2009). Despite this variability, SGs typically contain several essential elements. Among these are messenger RNAs (mRNAs), which accumulate in SGs when stress-induced translation stalls (Anderson and Kedersha, 2009). The regulation of this mRNA pool is facilitated by RNA-binding proteins (RBPs) such as T-cell intracellular antigen 1 (TIA-1), Poly-A-binding protein (PABP), Human antigen R (HuR) and Fragile X-related protein 1 (FXR1), which function in mRNA stability. The RBPs also play crucial roles in SG assembly through liquid–liquid phase separation (Fan and Leung, 2016). The translational components that appear universal to all SGs, independent of stress stimuli, include PolyA(+) mRNA, preinitiation factors such as eukaryotic translation initiation factor 4E (eIF4E), eukaryotic translation initiation factor 4G (eIF4G), eukaryotic translation initiation factor 3 (eIF3), Poly-A-binding protein 1 (PABP-1), and small ribosomal subunits. On the other hand, the SG-association of calcium-transporting ATPase (PMR1), eukaryotic translation initiation factor 2B (eIF2B), phosphorylation of eukaryotic initiation factor-2α (phospho-eIF2α), heat shock protein 70 (HSP70), and heat shock protein 27 (HSP27) varies depending on the type of cell and particular stressor (Anderson and Kedersha, 2002, 2008; Kedersha and Anderson, 2009). A variety of RNA-binding proteins, many of which can both bind and oligomerize RNA, can also promote SG assembly in addition to noncanonical 48S complexes. TIA-1/TIAR is one of these proteins (Gilks et al., 2004).

SG composition undergoes dynamic changes in response to stress duration and severity. In live cells, prolonged stress causes TDP-43-associated SGs to become excessively stable and form non-fluid gels (Wolozin and Ivanov, 2019). A time-resolved proteomic profiling of heat shock-induced SGs revealed that their protein composition changes during stress conditions (Hu et al., 2023). This indicates that the early-phase proteins of SGs are specifically enriched in proteins with RNA-related complexes, such as elements of the 40S ribosomal subunit, the C complex of the spliceosome, and the large Drosha complex, and have a solid phase-separation propensity. This is crucial for the rapid formation of the early SG structure. To further functionalise the SGs, late SG proteins such as eukaryotic initiation factor 3 (eIF3), the regulatory complex of the 26S proteasome (PA700), the profilin-1 complex, and the multisynthetase complex (MSC) are recruited later, suggesting a sequential recruitment process depending on the length of stress (Hu et al., 2023).

The protein composition of acute and chronic SGs varies, leading to distinct cellular outcomes. The composition of acute SGs encompasses signaling elements and 40S ribosomes that are highly dynamic and play a pro-survival role in the cell. Chronic stress forms more static SGs that lack 40S subunits and contain proteins that have a pro-death role in the cell (Reineke and Neilson, 2019). This change in protein composition highlights the importance of SGs in cellular adaptability in response to various stress conditions.

## Stress granules (SGs) and ALS

Under conditions of prolonged cellular stress *in vitro*, SGs may fail to disassemble efficiently, leading to disrupted RNA metabolism and impaired protein homeostasis (Dubinski and Vande Velde, 2025; Glauninger et al., 2022; Müller, 2021; Shelkovnikova et al., 2017). While SGs are typically transient and protective, they are associated with the pathology of neurodegenerative diseases, such as ALS and fronto-temporal dementia (FTD) (Aulas and Vande Velde, 2015; Li et al., 2013). Notably, multiple proteins associated with ALS, such as TDP-43, FUS, heterogeneous nuclear ribonucleoprotein A1 (hnRNPA1), and heterogeneous nuclear ribonucleoprotein A2/B1 (hnRNPA2B1), are recruited to SGs, and mutations in these proteins often result in aberrant SG dynamics (Wolozin and Ivanov, 2019; Xue et al., 2020). However, it is still unclear if SGs are a cause or consequence of neurodegeneration. Live-cell imaging systems show that SGs formed during stress may form persistent cytoplasmic aggregates that sequester RBPs and disrupt RNA metabolism (Hu et al., 2023). Determining the temporal aggregation of SGs and establishing if their presence is one of acute or chronic formation *in vivo* is challenging. The analysis of post-mortem tissue has identified SG-like structures containing aggregated RBPs, such as TDP-43, FUS, TIA-1, G3BP1 and eIF3, in ALS-affected MNs (Asadi et al., 2021). Our team recently analyzed the insoluble fraction isolated from the brain of late-stage rNLS TDP-43 ALS mice. The rNLS mouse model contains a doxycycline-inducible system designed to express the human TDP-43 protein with a deleted nuclear localization signal, causing its accumulation in the cytoplasm of neurones in the brain and spinal cord. Expression is driven by a human neurofilament promoter (Walker et al., 2015). Using liquid chromatography-mass spectrometry, an enrichment of 134 proteins was observed. Bioinformatic analysis of these proteins suggested that SG formation may influence MN degeneration via starvation-induced mitochondrial dysfunction (Dunlop et al., 2024). However, analysis of post-mortem human or late-stage disease mouse tissue provides only a single snapshot in time and does not allow for causal observations. Temporal analysis of SG formation and composition during disease onset and progression, in relevant mouse models, would provide insights into how acute or chronic SG formation and composition may influence disease-modifying mechanisms in ALS and other neurodegenerative diseases.

Dysregulation of SG dynamics, linked to impaired SG assembly, disassembly, and clearance, has negative consequences on the sequestration of RBPs and mRNAs into insoluble aggregates, impaired RNA metabolism, induction of chronic stress responses, translational repression, and facilitation of co-aggregation of other SG components, such as Ras GTPase-activating protein-binding protein 1 (G3BP1) and cell cycle associated protein 1 (caprin-1) (Baron et al., 2013; Dudman and Qi, 2020; Li et al., 2013). Furthermore, SG dynamics (assembly and disassembly) are regulated by post-translational changes such as ubiquitination, methylation, SUMOylation, acetylation, glycosylation, and phosphorylation (Ohn and Anderson, 2010; Wang et al., 2023). Interestingly, cellular dysfunction in ALS is also exacerbated by disturbances caused by these post-translational modifications.

## Myogranules (MGs) and their composition

MGs are transient, amyloid-like ribonucleoprotein (RNP) assemblies that form in regenerating skeletal muscle following injury. These cytoplasmic structures, typically 50–250 nm in size, contain RNA-binding proteins (RBPs) (Vogler et al., 2018). The most notable is TDP-43, which redistributes from the nucleus to the cytoplasm during muscle regeneration (Vogler et al., 2018). TDP-43 binds specific mRNAs encoding sarcomeric proteins, suggesting a scaffolding mechanism similar to SGs (Vogler et al., 2018). MGs are thought to facilitate the transport and translational repression of sarcomeric mRNAs near sites of new sarcomere formation, similar to neuronal mRNP transport granules. Sarcomeric proteins comprise approximately 60% of myofiber protein content and are essential for muscle function; abnormalities in their composition or organization can impair muscle performance (Cutler et al., 2019).

Experimental deletion of one allele of the Tardbp gene impairs muscle regeneration, underscoring the functional importance of TDP-43 in MG biology (Vogler et al., 2018). While the RNA–protein interaction mechanism is well-characterized, other mechanisms such as local translation regulation and phase transition dynamics may play a role in MG formation, although this remains to be experimentally validated (Vogler et al., 2018). Our understanding of MG formation and their role in health and disease is still emerging. To date, advanced imaging and biochemical techniques have assisted in characterizing their structure and clearance during muscle maturation (Vogler et al., 2018). Current research has focused narrowly on TDP-43, with limited exploration of other RBPs or RNA species. Furthermore, studies are largely based on mouse models, which questions relevance to human muscle pathology (Cutler et al., 2019). Further research is needed to elucidate the molecular triggers of MG formation, their regulation, and their potential transition to pathological aggregates in neuromuscular diseases.

MGs are typically eliminated from muscle cells within 10 days after injury as myofibers mature (Cutler et al., 2019; Vogler et al., 2018). However, if these aggregates persist, they may trigger the development of TDP-43-associated amyloid fibrils. Notably, mouse models of inclusion body myopathy (IBM) have similar pathogenic TDP-43 aggregates, pointing to a possible connection between chronic muscle degeneration and impaired MG clearance. It has therefore been proposed that the formation (increase assembly) or removal (decrease clearance) of functionally normal MG structures in muscle could affect the localization of TDP-43, potentially leading to cytoplasmic aggregate formation associated with neuromuscular disease (Vogler et al., 2018).

## Potential relationship between myogranules (MGs), stress granules (SGs) and ALS pathogenesis

**Figure 1:**
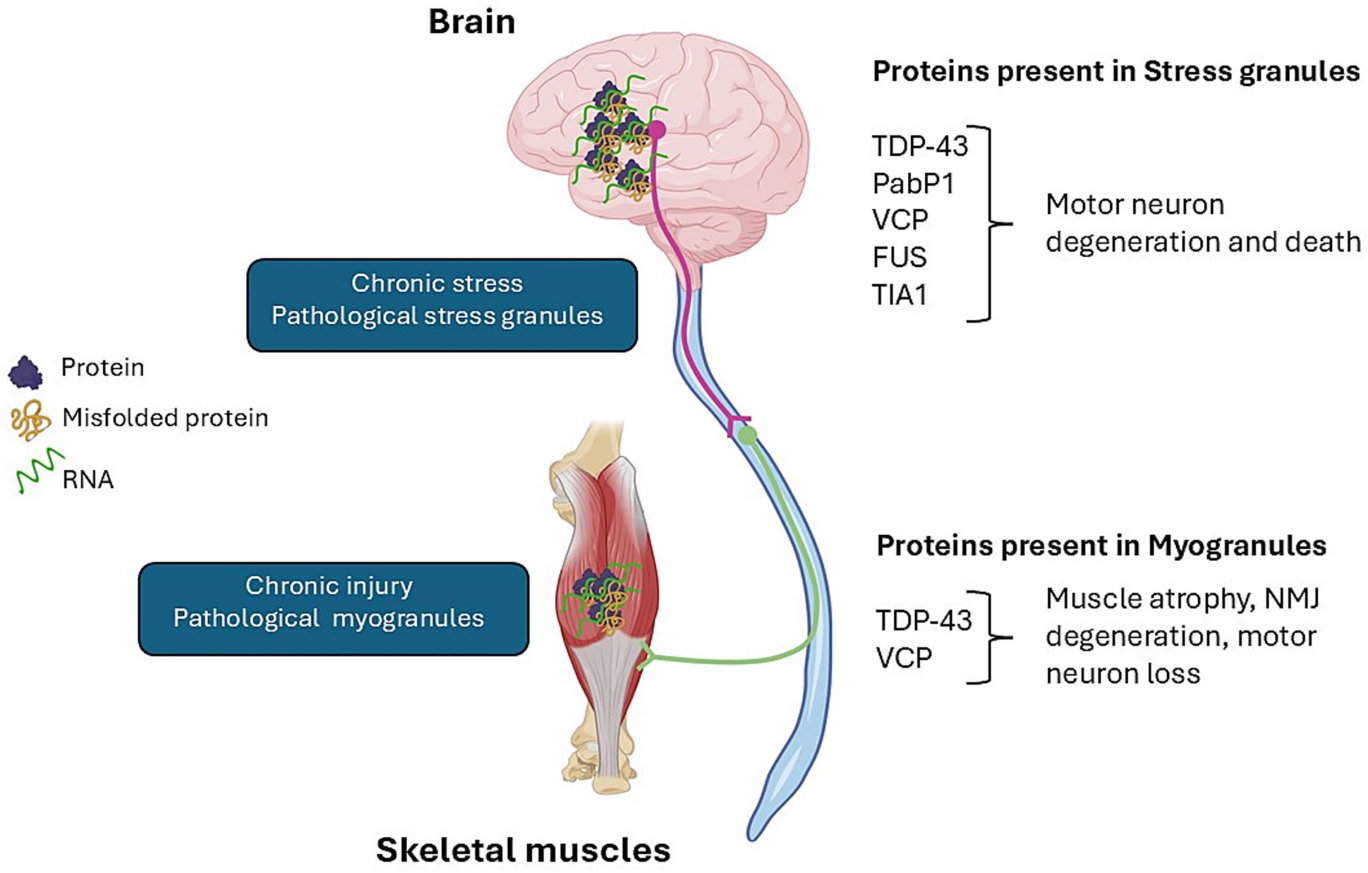
Proteins present in stress granules and myogranules and their potential role in neurodegeneration and muscle impairment. Created in BioRender. Russell, A. (2025) https://BioRender.com/vaux7yy.

The relationship between MGs and ALS remains unclear, but emerging evidence suggests a potential pathological link through shared protein components with SGs (Figure 1). At a late-stage in the rNLS mouse model of ALS, TDP-43 accumulates in skeletal muscle and colocalizes with *β*-amyloid markers in angular, atrophied, and denervated fibers, indicating the presence of TDP-43-associated granules (Tsitkanou et al., 2022). Given that amyloid assemblies are considered inherently pathological (Iadanza et al., 2018), this supports the hypothesis that skeletal muscle may contribute to ALS onset and progression. Whether ALS-linked TDP-43 mutations promote stable, gel-like inclusions within MGs, potentially marking an early step in pathological aggregation, remains to be investigated (Vogler et al., 2018).

Although MG composition is less defined than SGs, both granule types may share components and regulatory mechanisms related to RNA metabolism and stress responses. Several SG-associated proteins, including VCP, PABP1, TIA1, FUS, and Staufen1, have also been identified in skeletal muscle (Anderson and Kedersha, 2009; Asadi et al., 2021; Mahboubi and Stochaj, 2017; Riggs et al., 2020). Whether these proteins are found in MGs has not been determined. Investigating SG and MG proteomes across ALS disease stages may reveal novel mechanisms driving disease onset, progression, and severity. Below is a summary of these SG-related proteins found in skeletal muscle and their potential role in ALS (Table 1). Understanding the role and regulation of these SG-related proteins in skeletal muscle may help to better understand the pathological role skeletal muscle plays in ALS pathogenesis.

**Table 1:** An overview of the SG and MG proteins, as well as a brief description of their role in the brain and/or skeletal muscle.

Protein	Granule	Function in brain	Function in muscle
Pabp1	SG	Binds to mRNA poly(A) tails, controls translation and stability, and localises to SGs under stress.	Crucial for a number of processes including RNA processing, muscle development, and cytoskeletal integrity; found in stress granules.
TIA1	SG	Regulates alternative splicing and promotes stress granule assembly.	Contributes to the integrity of muscles; mutations affect the removal of SG, which causes toxicity in the muscles.
FUS	SG	Involved in the regulation of RNA metabolism, including alternative splicing, transcription and stability; its mutation can influence neuronal function.	Critically involved in the differentiation of muscle and structural integrity. Mutation in FUS is associated with ALS.
Stau1	SG	Involved in the regulation of neuronal differentiation, synaptic plasticity and mRNA transport.	Plays a complex role that involves the regulation of RNA metabolism and influences muscle growth, differentiation and development.
VCP	SG/MG	Crucially takes part in the clearance and quality control of proteins. Mutations in VCP are linked to ALS.	Takes part in the clearance and quality control of proteins. Mutations in VCP are linked to generative disease such as inclusion body myopathy.
TDP-43	SG/MG	Plays a role in gene expression regulation, pre-mRNA splicing, RNA stability. Take part in the formation of stress granules. TDP-43 mutations are linked to ALS.	Plays a vital role during muscle regeneration. Takes part in the formation of SGs and MGs during stress response and during muscle regeneration. Mutation in TDP-43 is linked to ALS.

## Valosin-containing protein (VCP)

Valosin-containing protein (VCP), commonly referred to as P97, is a conserved member of the AAA-ATPase family and plays a crucial part in the clearance and quality control of proteins (Rabinovich et al., 2002; Shih and Hsueh, 2016). VCP is associated with both SGs and MGs (Seguin et al., 2014; Vogler et al., 2018). VCP mutations are associated with ALS (Johnson et al., 2010) and inclusion body myopathy with Paget disease and frontotemporal dementia (IBMPFD) (Vesa et al., 2009). Muscle degeneration and other disease-related symptoms are exacerbated by VCP mutations, which result in cellular stress reactions. In C2C12 myoblasts, after exposure to oxidative stress induced by arsenite, mutant VCP delays the cellular SG resolution (Rodriguez-Ortiz et al., 2016). This delay may contribute to chronic SG accumulation and cause cellular dysfunction and protein malfunction, which are common features of degenerative diseases (Moon, 2022).

## PolyA binding protein 1 (PABP1)

Poly(A)-Binding Protein 1 (PABP1) is a key protein in stress granule dynamics, playing a vital role in multiple facets of mRNA regulation, including translational initiation, degradation and stability (Kahvejian et al., 2005; Smith and Gray, 2010). In skeletal muscle, PABP1 is also crucial for muscle development, cytoskeletal integrity and RNA metabolism (Banerjee et al., 2017; Olie et al., 2020); the latter via its interaction with RNA-binding proteins, such as Matrin 3 (Banerjee et al., 2017).

Muscle atrophy is a result of impaired myogenesis, and disturbed cytoskeletal architecture caused by decreased PABP1 levels during muscle differentiation. Restoring PABP1 function enhances sarcomeric protein production and muscle cell fusion, both of which are essential for muscle structure and function (Olie et al., 2020). Reduced PABP1 levels during muscle differentiation cause cytoskeletal disruption and impaired myogenesis, which exacerbate muscle atrophy. Sarcomeric protein expression and muscle cell fusion, both of which are essential for muscle structure and function, can be enhanced by restoring PABP1 activity (Banerjee et al., 2013; Olie et al., 2020).

PABP-1 relates to ALS through its involvement in mediating the toxicity of TDP-43 in model systems, and its inclusions in the ALS spinal cord were also discovered. In various tissues from people with ALS, PABP-1 colocalizes with mature TDP-43 inclusions. Inclusions of PABP-1 can be circular or skein-like, depending on the ALS subtype. This presence indicates its common pathogenic function in ALS (Kim et al., 2014; McGurk et al., 2014).

## T-cell restricted intracellular antigen-1 (TIA1)

TIA1 is a ubiquitously expressed protein essential for muscle cell function through its participation in RNA metabolism, SG formation and dynamics, and involvement in muscle-related disorders (Alcalde-Rey et al., 2024). In addition to SG formation, mutations in TIA1, such as p. E384K, are observed in Welander distal myopathy, a classic adult-onset autosomal dominant disorder (Klar et al., 2013). Splicing of survival of motor neurone 2 (SMN2) at exon 7 in people with Welander distal myopathy is associated with a decrease in TIA1 in skeletal muscle and increased TIA1 staining linked to SGs. This suggests a role for TIA1 and RNA metabolism in maintaining the homeostasis of skeletal muscle (Klar et al., 2013). Crucially, TIA1 mutations or variations are linked to a myodegenerative phenotype. The TIA1-N357S variation inhibits SG dynamics in living cells and dramatically improves liquid–liquid-phase separation *in vitro*. TIA1-N357S-persistent SGs are more likely to associate with sequestosome-1 (SQSTM1), accumulate ubiquitin conjugates, and aggregate other proteins. Myoblasts with TIA1-N357S variant expression and an SQSTM1-A390X mutation have impaired SG clearance and increased myotoxicity (Lee et al., 2018). Moreover, TIA1 mutations in live cells are implicated in ALS pathogenesis through delaying the disassembly of SGs and helping in the accumulation of non-dynamic SGs that harbor TDP-43 (Mackenzie et al., 2017).

## Fused in sarcoma (FUS)

FUS recruits transcription factors, including myocyte enhancer factor 2 (MEF2) and ETS variant transcription factor 5 (ETV5), through liquid–liquid phase separation to promote muscle growth. It also controls the transcription of MEF2-dependent genes essential for muscle differentiation (Picchiarelli et al., 2024). FUS also preserves the structural integrity of muscles, while FUS mutations linked to ALS result in ultrastructural abnormalities, such as damaged mitochondria and sarcomeres (Picchiarelli et al., 2024). Interestingly, FUS functions as a repressor of myogenesis by sequestering troponin T1 (Tnnt1) mRNA to the nucleus. Myogenesis is promoted when FUS levels are decreased, as it enables Tnnt1 mRNA to be exported to the cytoplasm (Ji et al., 2024). FUS controls the gene expression of acetylcholine receptors in subsynaptic muscle cell nuclei, and the harmful consequences of ALS-related FUS mutations in muscle cells could contribute to neuromuscular junction impairments and retrograde MD death (Picchiarelli et al., 2019). In ALS, mislocalized FUS is associated with mitochondrial abnormalities that lead to muscle degeneration, such as disorganized cristae and swelling (Yu et al., 2022).

## Staufen1 (STAU1)

As a member of the dsRNA-binding protein (dsRBP) family, STAU1 mediates mRNA transport and localisation and controls mRNA processing, stability, and translational activity (Yadav et al., 2020). STAU1 is abundantly expressed in embryonic stages of muscle development, decreasing as muscle matures. It upregulates atrophy-related genes and inhibits the PI3K/AKT signaling pathway, which is critical for muscle growth (Crawford Parks et al., 2017; Ravel-Chapuis et al., 2014). STAU1 overexpression also increases c-myc, which impedes muscle cell differentiation. This demonstrates that healthy muscle growth and maintenance require precise STAU1 control (Ravel-Chapuis et al., 2014).

In myotonic dystrophy type 1 (DM1), STAU1 increases considerably, where it facilitates the translation and nuclear export of mutant mRNAs. Additionally, it regulates alternative splicing, which aids in reversing the abnormal splicing patterns observed in DM1. These results imply that STAU1 might be a viable target for treatment in neuromuscular disorders that cause muscle dysfunction.

In conclusion, the potential trapping of similar proteins in both SGs and MGs suggests a pathological connection between stress responses in neuronal tissue and skeletal muscle. These proteins, which are normally involved in dynamic RNA control during cellular stress, may lose functional mobility and lead to their persistent abnormal aggregation. This mislocalization may disrupt normal RNA metabolism and contribute to the formation of toxic protein inclusions, not only in the brain and spinal cord, but also in skeletal muscle. The trapping of SG-associated components in MGs may be a shared mechanism of cellular dysfunction across tissues, linking impaired granule dynamics to broader ALS pathology. Determining the temporal protein composition in both SGs and MGs throughout disease progression will elucidate the relationship, if any, between these granules. This may also assist with better understanding their role/s, either pathological or protective, in neurological disease.
